# Regulation of T cells by myeloid-derived suppressor cells: emerging immunosuppressor in lung cancer

**DOI:** 10.1007/s12672-023-00793-1

**Published:** 2023-10-19

**Authors:** Zhong-Ning He, Chun-Yu Zhang, Yu-Wei Zhao, Shu-Lin He, Yue Li, Bo-Lun Shi, Jia-Qi Hu, Run-Zhi Qi, Bao-Jin Hua

**Affiliations:** 1 Guang’anmen Hospital, China Academy of Chinese Medical Sciences, Beijing, China; 2 Shanxi Medical University, Shanxi, China

**Keywords:** Myeloid- derived suppressor cells, T cells, Lung cancer, Immune checkpoint inhibitors, Immunity regulation

## Abstract

Myeloid-derived suppressor cells (MDSCs), major components maintaining the immune suppressive microenvironment in lung cancer, are relevant to the invasion, metastasis, and poor prognosis of lung cancer, through the regulation of epithelial-mesenchymal transition, remodeling of the immune microenvironment, and regulation of angiogenesis. MDSCs regulate T-cell immune functions by maintaining a strong immunosuppressive microenvironment and promoting tumor invasion. This raises the question of whether reversing the immunosuppressive effect of MDSCs on T cells can improve lung cancer treatment. To understand this further, this review explores the interactions and specific mechanisms of different MDSCs subsets, including regulatory T cells, T helper cells, CD8 + T cells, natural killer T cells, and exhausted T cells, as part of the lung cancer immune microenvironment. Second, it focuses on the guiding significance confirmed via clinical liquid biopsy and tissue biopsy that different MDSC subsets improve the prognosis of lung cancer. Finally, we conclude that targeting MDSCs through action targets or signaling pathways can help regulate T-cell immune functions and suppress T-cell exhaustion. In addition, immune checkpoint inhibitors targeting MDSCs may serve as a new approach for enhancing the efficiency of immunotherapy and targeted therapy for lung cancer in the future, providing better comprehensive options for lung cancer treatment.

## Introduction

Various types of immune responses, including those in cancer, involve T cells, such as CD8^+^ T cells and CD4^+^ T helper (Th)1 cells. These T cells act as the main immune cells against cancer [1]. Particularly, CD8 + T cells are the main targets of immune checkpoint inhibitors (ICIs) within the immune microenvironment. Myeloid-derived suppressor cells (MDSCs) are major immune precursor cells that influence T-cell differentiation and immune function either directly or indirectly through interactions with T cells during the formation of the immunosuppressive microenvironment of a tumor, thus maintaining the immunosuppressive microenvironment of lung cancer. MDSCs mediate the epithelial-mesenchymal transition (EMT) and regulate the invasion and metastasis of lung cancer. They also weaken the efficacy of T-cell ICIs through immune regulation, which is an obstacle in immunotherapy [2]. The incidence and mortality rates of lung cancer are among the highest in the world [3], and a large amount of MDSCs often accumulate in the peripheral blood and tumor tissues of patients with lung cancer [4]. With the stage of lung cancer increasing over time, the degree of infiltration of MDSCs into the immune microenvironment of tumor tissues gradually increases, and the rise in the number of MDSCs is related to the degree of immunosuppression and a poor prognosis [5, 6]. Other suppressive or regulatory cells can be recruited and induced by MDSCs, such as regulatory T (Treg) cells, which inhibit the immune function of various T-cell types, including natural killer T (NKT) and CD8^+^ T cells, through multiple pathways. MDSCs can encourage the depletion of CD8^+^, CD4^+^, and memory T cells (T-cell exhaustion), thus affecting the immune function of patients with tumors. These findings raise the question of whether reversing the immunosuppressive effect of MDSCs on T cells can lead to improved lung cancer treatment. To understand this further, this study reviews recent research on the interactions and specific mechanisms of different MDSCs subsets, including Treg, Th, CD8^+^ T, NKT, and exhausted T cells, as part of the lung cancer immune microenvironment. The review also focuses on the guiding significance confirmed via clinical liquid biopsy and tissue biopsy that different MDSC subsets improve the prognosis of lung cancer.

## Phenotype of MDSCs

MDSCs are heterogeneous immature bone marrow cell (IMC) populations at different stages of differentiation. Normally, IMCs differentiate into mature granulocytes, monocytes, and dendritic cells (DCs), after they migrate to peripheral organs. However, within the tumor microenvironment (TME), tumor cells and their targets such as STAT3, IRF8, C/EBPβ, and Notch can inhibit the differentiation of IMCs. Specific factors produced by activated T cells, tumor, and tumor stromal cells, such as VEGF, CSF, IL-6, IL-10, MMP-9, TGF-β, NF-kB, and other immune-suppressive mediators, can stimulate the proliferation and activation of MDSCs [7, 8]. Activated MDSCs selectively accumulate in the peripheral blood and tumor tissues from patients with cancer and exert immunosuppressive activity [8].

Animal experiments have shown that MDSCs express different surface antigens in animals and humans. In 2010, Dolcetti proposed that MDSCs can be divided into two subgroups—monocytic MDSCs (M-MDSCs) and polymorphonuclear MDSCs (PMN-MDSCs)—and confirmed that MDSCs are a heterogenous population of cells that can be dissolved using Ly6C and Ly6G in mice [9]. MDSCs express the membrane antigens CD11b and Gr1 in mice and can be subdivided into two subpopulations based on different Gr1 epitopes: CD11b^+^Ly6G^+^Ly6C^low^ and CD11b^+^Ly6G^−^Ly6C^high^ [10–12]. Haverkamp JM demonstrated that MDSCs can be defined independently of their phenotype [13]. In humans, MDSCs are of mainly two types: M-MDSCs and PMN-MDSCs, according to their differentiation. PMN-MDSCs are defined as CD11b^+^CD14^−^CD15^+^ or CD11b^+^CD14^−^CD66b^+^, whereas, M-MDSCs are defined as CD11b^+^ CD14^+^ CD15^−^ HLA^−^ DR^−/low^. Bronte et al. proposed naming a mixed group of MDSCs consisting of a greater number of immature progenitor cells with the phenotype of early stage MDSCs (e-MDSCs) [10, 12] (Table 1).

**Table 1:** Phenotype of MDSCs

Subset	Phenotype (Mouse)	Phenotype (Human)
M-MDSCs	CD11b^+^Ly6G^−^Ly6C^high^	CD11b^+^ CD14^+^ CD15^−^ HLA^−^ DR^− /low^
PMN-MDSCs	CD11b^+^Ly6G^+^Ly6C^low^	CD11b^+^CD14^−^CD15^+^
		CD11b^+^CD14^−^CD66b^+^
e-MDSCs	–	Lin^−^HLA^−^DR^−^CD33^+^

## Significance of MDSCs in patients with lung cancer

Single-cell RNA-seq, flow cytometry, and cell mass spectrometry have been used to determine the heterogeneity of polymorphonuclear neutrophils (PMNs) in patients with lung cancer. PMN-MDSCs in such patients resemble activated PMN-MDSCs in mice in terms of genetic characteristics and have negative prognostic outcomes in the patients [14]. With the increasing number of clinical histopathology and liquid biopsy studies, previous studies on the phenotypes of MDSCs in tumor tissues or peripheral blood from patients with non-small cell lung cancer (NSCLC) and small cell lung cancer (SCLC) have been diverse, as shown in Table 2. Despite the different phenotypes studied, the percentage of MDSCs in the tumor tissues and peripheral blood of patients with lung cancer is found to be increased, compared with that in healthy people. With an increase in the stage of lung cancer, the degree of MDSC infiltration into the tumor tissue immune microenvironment gradually increases. Compared with early stage patients, advanced stage patients have higher concentrations of MDSCs present in their peripheral blood [5], and increased numbers of MDSCs are linked with the degree of immunosuppression and a poor prognosis [6]. Patients with high MDSCs infiltration concentrations tend to have a shorter overall survival, lower survival rates, and poorer prognoses [15–18]. Kohsuke used single-cell level 16- or 17-color multiplex immunohistochemistry (mIHC) spatial analysis to assess the tumor immune microenvironment in patients with advanced NSCLC resistant to treatment with ICIs. The results demonstrated that resistance to ICIs was associated with a substantial infiltration of MDSCs and M2 type tumor-associated macrophages in the TME [19]. The immune function of lung cancer patients can be inhibited by a large amount of infiltration by MDSCs, promote disease progression and induce distant metastasis [20, 21]. In patients with elevated levels of MDSCs,their peripheral blood often exhibits resistance to multiple therapies such as immunotherapy, targeted therapy, and chemotherapy [22–25] (Table 2).

**Table 2:** Clinical significance of MDSCs with different phenotypes

Lung cancer types	Sample	Phenotype of MDSCs	Clinical significance	Refs.
NSCLC	Peripheral blood	CD14^+^HLA^−^DR^−^(M-MDSC)	Upregulation of M-MDSC was associated with shorter Overall Survival (OS) and shorter Median Survival (MST)	[15]
NSCLC	Peripheral blood	CD66b^+^CD11b^+^CD15^+^CD14^−^(PMN-MDSC)	Elevated PMN-MDSC suggests decreased OS	[16]
NSCLC	Peripheral blood	CD14^+^CD15HLA^−^DR^−^Lin^−^(M-MDSC)	Elevated M-MDSC indicates poor tumor staging, poor first-line treatment, and reduced Progression Free Survival (PFS) and OS	[17]
NSCLC	Peripheral blood	Lin-CD15 + CD14-CD11b + HLA-DR^−/Low^ (PMN-MDSC), Lin^−^CD15^−^CD14^+^HLA^−^DR^−/Low^(M-MDSC)	Elevated PMN-MDSC and M-MDSC suggest poor tumor stage, reduced PFS and OS, and poor PD-1 treatment	[5]
NSCLC	Tumor tissue, peripheral blood	HLA^−^DR^−/low^CD11b^+^CD14^−^CD15^+^(PMN-MDSC)	Elevated PMN-MDSC suggests reduced Recurrence Free Survival (RFS), and the frequency of circulating and tumor-infiltrating PMN-MDSC increases with tumor progression	[4]
NSCLC	Peripheral blood	CD11b^+^CD14^+^S100A9^+^	Elevated CD11b^+^CD14^+^S100A9^+^ MDSCs suggest poor chemotherapy efficacy, reduced PFS, and shorter Median Survival Time (MST)	[159]
NSCLC	Peripheral blood	CD11b^+^CD14^+^HLA^−^DR^−^CD33^+^CD15^+^(Mo-MDSCs), CD11b^+^CD14^−^HLA^−^DR^−^CD33^+^CD15^+^(PMN-MDSC)	Patients with elevated Mo-MDSCs, PMN- MDSCs, decreased OS	[18]
NSCLC	Peripheral blood	CD14^+^HLA^−^DR^−^(Mo-MDSCs)	Accumulation of Mo-MDSCs was associated with poor tumor stage, decreased OS, short chemotherapy cycles, decreased lymphocyte to monocyte ratio, and decreased mean platelet volume to platelet count ratio	[160]
NSCLC	Peripheral blood	B7^−^H3^+^CD14^+^HLA^−^DR^−/low^	Reduced RFS in patients with elevated B7^−^H3^−^MDSC is associated with poor prognosis	[161]
Advanced lung adenocarcinoma	Tumor tissue	CD45^+^CD11b^+^Ly6G^hi^	Upregulation of MDSCs predisposes to EMT metastatic lesions	[20]
NSCLC	Peripheral blood	CD33^+^CD11b^+^HLA^−^DR^low^	High incidence of brain metastases and reduced OS in upregulated MDSCs	[21]
SCLC	Peripheral blood	CD33^+^CD11b^+^HLA^−^DR^−^	Elevated CD33^+^CD11b^+^HLA^−^DR^−^MDSCs suggest poor tumor staging, metastasis-related and poor treatment efficacy	[162]
SCLC	Peripheral blood	CD14^+^HLA^−^DR^−/low^	The accumulation of CD14^+^HLA^−^DR^−/low^ MDSCs suggested poor tumor staging, decreased serum LDH levels, and poorer OS	[163]
NSCLC	Peripheral blood	CD14^+^HLA^−^DR^−/low^(Mo-MDSCs)	Elevated Mo-MDSCs are associated with tumor susceptibility to metastasis, poor chemotherapy efficacy, and reduced PFS	[164]
Postoperative lung cancer	Peripheral blood	CD11b^+^CD33^+^HLA^−^DR^−^CD14^+^(M-MDSC)	Elevated M-MDSC is associated with reduced RFS and higher levels of T-cell suppression	[165]
EGFR mutant lung adenocarcinoma	Peripheral blood	CD11b^+^CD14^+^S100A9^+^	Elevated S100A9^+^MDSC is associated with poor EGFR-tyrosine kinase inhibitor (EGFR-TKI) treatment efficacy and reduced PFS	[22]
Metastatic NSCLC	Peripheral blood	CD14^+^CD15^+^HLA^−^DR^−^CD33^+^(M-MDSC)	Elevated M-MDSC predisposes to primary and secondary resistance to PD-1 therapy	[23]
NSCLC	Peripheral blood	CD14^+^HLA^−^DR^−^(M-MDSCs)	Elevated M-MDSCs are associated with immunotherapy resistance, systemic inflammatory response, and decreased PFS and OS	[24]
NSCLC	Peripheral blood	CD11b^+^CD14^−^CD33^+^	Patients with upregulated MDSCs had poor efficacy of systemic chemotherapy, reduced proportion of CD8^+^T cells, and suppressed immune function	[25]

MDSCs and their specific molecules, such as TIE2^+^ [15], S100A9^+^ [22], PD-L1, and CCR5 [4], can be used as predictive markers for the recurrence and metastasis of lung cancer, and as monitoring indicators to judge the efficacy of chemotherapy, targeted therapy, and immunotherapy. MDSCs exert immunosuppressive effects by inhibiting T-cell function. Therefore, identifying the mechanism of the interaction between MDSCs and T cells and targeting MDSCs to inactivate them and reverse the failure of T cells and the treatment of lung cancer is dependent upon the body's immune function.

## Interaction of MDSCs with T cells

Activation of MDSCs is the major cause of lung cancer immune suppression. The mechanism of MDSC-mediated immunosuppression is closely related to the dysregulation of T-cell immune functions, and the mechanism by which MDSCs inhibit T-cell immune functions can be summarized as *follows: (1) The immune function of T cells is inhibited by MDSCs by affecting the amino acid metabolism. MDSCs* have been shown to affect amino acid metabolism in T cells; they consume arginine, cysteine, tryptophan, and other essential amino acids necessary for T-cell proliferation and activation; and they affect T-cell immune function. MDSCs deplete L( +)-arginine, which plays a critical role in T-cell activation and metabolism in the immune microenvironment, T-cell receptor (TCR) CD3ζ chain downregulation and signaling is due to a high expression of arginase 1 (ARG1) by the MDSCs, and T-cell dysfunction. In addition, L ( +)-arginine depletion prevents T cells from entering the G1 phase by inhibiting cyclin D3 expression, which induces T-cell cycle arrest [26, 27]. Cysteine is an indispensable amino acid for T-cell activation and function. Because T cells have a cysteine transporter defect, they can only obtain cysteine from antigen-presenting cells (APCs) such as macrophages and DCs, whereas MDSCs are known to compete with APCs for extracellular cysteine and do not export cysteines. It thereby prevents T-cell proliferation and activation [28]. MDSCs degrade the tryptophan required for T-cell proliferation to N-formyl epinephrine by overexpressing indoleamine 2,3-dioxygenase (IDO). The lack of tryptophan leads to the arrest of the T-cell cycle [29], which inhibits T-cells immune function. *(2) MDSCs inhibit T cells immune functions by secreting a variety of reactive oxygen species and immunosuppressive factors.* MDSCs express various enzymes involved in the production of reactive oxygen species (ROS) and nitric oxide (NO). The production of MDSCs induces the upregulation of Arg-1 activity and ROS production in a pathway dependent on Signal Transducers and Activators of Transcription 3 (STAT3). Indicating that the STAT3 activator signal plays a critical role in MDSC functions. Peroxynitrite (the product of ROS reacting with NO) alters TCR and CD8 molecules, and deprives cytotoxic T lymphocytes (CTLs) of the ability to specifically bind to the major histocompatibility complex (MHC) class I molecules, leading to further loss of their ability to kill tumor cells [30–33]. In addition, inducible NO synthase (iNOS) expressed by MDSCs can produce large amounts of NO, which blocks both the phosphorylation and the subsequent activation of IL-2 receptor-related proteins. NO also reduces the stability and release of IL-2 mRNA, further blocking the activation of T cells and inhibiting their proliferation [31, 34]. *(3) MDSCs inhibit T-cell immune functions by elevating PD-L1 receptor expression.* It is well known that the tumor immune microenvironment often presents an oxygen-deficient state, and in this hypoxic TME, hypoxia-inducible factor-1α (HIF-1α) is highly expressed. HIF-1α can induce programmed death ligand-1 (PD-L1) expression on the surface of MDSCs by binding to the programmed death factor-1 (PD-1) on the T-cell surface, which causes T-cell immune dysfunction and induces T-cell depletion and apoptosis [35, 36].

### Immune regulation of Tregs by MDSCs

Tregs are a subgroup of CD4^+^ T lymphocytes cells that express CD25 and Foxp3 (CD4^+^CD25^+^ FoxP3^+^) [37], and are known for their modulatory effects on the T cell-killing function. Tregs exert a dual regulatory effect on T-cell autoimmunity. When Tregs inhibit T-cell death function, T cells display immune inertia, and the recognition of alloantigens and the production of cytotoxic particles and killer factors are weakened. When Tregs are known to promote T-cell immune activity, T cells will show immune hyperactivity and release excessive cytotoxic particles and killing factors, leading to the abnormal recognition of antigens, and even the killing of autologous cells. In the tumor immune microenvironment, owing to their ability to inhibit autogenic responses, Treg cells may impede antitumor immune responses. Moreover, specific depletion or functional changes in Treg cells can experimentally evoke effective tumor immunity [38]. Previous clinical studies have confirmed that a higher proportion of Tregs are found in the peripheral blood of patients with lung cancer [39, 40]. Tregs are key to the immune escape of lung cancer cells and are correlated with the progression and distant metastasis of lung cancer [41, 42]. At the same time, MDSCs and Tregs, two important immune cell populations in the immune microenvironment, also interact with each other. Granulocytes become sensitive to hypoxic conditions and are more likely to adopt immunosuppressive behaviors toward T cells [35]. Mark et al. found that in high-grade tumors, the immunosuppressive capacity of neutrophils is enhanced, and there is a tendency to interact with Treg cells [43]. MDSCs Isolated from in the peripheral blood of tumor-bearing mice have been shown to express elevated levels of chemokines such as CCL3, CCL4, and CCL5 [44], and the production of these chemokines is closely related to the recruitment of Tregs [44, 45]. Adoptive transfer experiments showed that MDSCs can induce Treg recruitment and generation and inhibit the antitumor T-cell response, while Treg cell content decreased significantly after treatment with anti-IL-10 or anti-IFN-γ [46], In a study conducted by Pan, the levels of IL-10 and TGF-β secreted by MDSCs were found to be significantly increased under IFN-γ stimulation, and the recruitment and production of Tregs also increased. Anti-CD40 was applied to inhibit CD40 expression on the MDSC surface and reduce the specific binding of MDSCs to the CD40 ligand (CD40L) expressed on Tregs, resulting in the reduction of the level of Treg expansion [47]. The application of IL-2/anti-CD40 effectively reduces the levels of Tregs and Treg-related chemokines in mice with tumors [48]. IL-10 and IFN-γ, as well as the expression of CD40 on the MDSC surface, are necessary for the development and expansion of Tregs. Furthermore, immunosuppressive factors like Arg1, NO, IL-10, and TGF-β produced by MDSCs can participate in the recruitment of Tregs by upregulating HIF-1α protein [49]. IL-10 can induce CD4^+^ T cells to express Foxp3 and increase the number of Tregs [50, 51]. It can also enhance the differentiation of Tregs and to promote their immune suppressive functions by promoting STAT3 phosphorylation [52]. TGF-β released from MDSCs can induce the expression of CD25 and Foxp3, and convert naive CD4 + T cells into Tregs [53, 54].

### Interaction between MDSCs and Th cells

Th cells are different subsets formed from naive CD4 + cells upon activation. They produce cytokines and play important roles in immune regulation via the secreted cytokines. They can be divided into Th1, Th2, and Th17 cells, depending on the specific cytokines that they produce. Th1 cells regulate viruses, bacterial infections, and the development of specific autoimmunity and secrete vital cytokines such as INF-α, IFN-γ, and IL-12. Their immune action is mainly achieved through the activation of immune cells such as macrophages, CD8 + T, and NK cells, to mediate protective immune responses within the TME [55, 56]. The Th1/Th2 cell ratio differs in different diseases, and the proportion of Th2 cells in cancer patients is relatively high [57]. Additionally, Th2 cells mediate reactions such as parasitic infection, inflammation, and allergy, by promoting the humoral immune response and secreting cytokines such as IL-4, IL5, IL-10, and IL-13, which not only inhibit the Th1 cell immune tumor infiltrating T-cell response but also benefit Treg development. A large amount of Th2 aggregation is associated with the tumor escape mechanism [58]. Finally, Th17 cells are mainly responsible for bacterial-fungal infections and some autoimmune responses [58, 59]. However, the role of Th17 cells in the lung cancer immune microenvironment remains controversial. Martin-Orozco [60] found that Th17 cells can promote the recruitment of T cells to tumor sites, participate in antitumor immunity, and activate the production of CCL2 and CCL20 by secreting IL-17-activated border cells. These chemokines mobilize dendritic cells (DCs) and other leukocytes to the site of the tumor and further activate tumor-specific CD8^+^ T cells. Immunohistochemical tests among lung cancer patients showed that a large amount of immunosuppressive CD4^+^ T cells, including Th2 cells and Tregs, accumulated in the epithelium and stroma, whereas only a small number of Th1, Tfh, and Th17 cells were observed [61]. However, most studies have suggested that Th17 cells negatively affect the immune function in lung cancer patients. In lung cancer cells of mice, Th17 lymphocytes induce EMT, thereby promoting the migration and diffusion of metastases [62]. Peng et al. found that Th17 cell infiltration is linked to treatment resistance in patients with lung cancer. Th17 cells can secrete IL-17 and IL-22, which promote lung cancer cell metastasis and MEK inhibitor resistance [63]. In another study, the density of IL-17 producing cells in the tumor stroma was reported to be negatively correlated with the survival of NSCLC patients [64].

Promotive and inhibitory regulatory relationships exist among MDSCs and Th cells within the lung cancer TME. A mutual inhibitory relationship between MDSCs and Th1 cells has been reported. Coculture experiments have found that Th1 produces cytokines such as IL-2 and IFN-γ, which are capable of reducing the number of MDSCs and inhibiting their function by inducing the production of chemokines like CXCL9 and CXCL10, and reducing the levels of immune factors like IL-6 and IL-1β that promote MDSC proliferation and activation [65]. Yu et al. found that IDO expression in MDSCs isolated from tumor tissues was significantly upregulated and that MDSCs inhibited the polarization of Th1 cells by promoting IDO expression [66]. IFN-γ secreted by Th1 cells is an important cytokine that plays a significant role in inhibiting and killing tumor cells, as well as retarding the growth of tumors. TGF-β, secreted by MDSCs, is an immunosuppressive cytokine that is a key player in carcinogenesis and tumor progression. According to some studies, different mechanisms are used by TGF-β to inhibit the expression of IFN-γ in CD4^+^ Th1 cells at the time of initiation and recall [67], which then block the body’s autoimmune response to promote tumor growth and progression [68]. Additionally, with a reduction in MDSCs infiltration in the TME, Th1 polarization of CD4 + T cells increased, as did the cytotoxicity of CD8 + T cells [69]. In vivo and in vitro experiments have confirmed that MDSCs can promote the differentiation of Th2 cells, which may be achieved by the Th2-related immunosuppressive molecule IL-13 [70, 71]. Coculture experiments with MDSCs and Th17 cells showed that MDSCs promoted Th17 cell transformation into Tregs by secreting TGF-β and retinoic acid (RA). Thus, blocking RA or TGF-β can reduce the transformation of Th17 cells into Tregs [72].

### CD8^+^ T-cells immune function is inhibited by MDSCs

Naive CD8^+^ T cells, after activation by APC and recognition by the TCR of peptide-specific MHC-I complexes, activate and differentiate into effector CTLs, which are then capable of targeting and killing cells (such as infected cells and cancer cells) and secreting protective cytokines [73]. In the TME, two key processes are involved in the antitumor effects of CD8^+^ T cells: migration and differentiation [74, 75]. Upon entry into the TME, the initial population of CD8^+^ T cells differentiates into effector CD8 + T cells, which are then activated and further differentiated into cytotoxic and memory CD8^+^ T cells [75, 76], During this process, transcription factors from the internal and external environments regulate the expression of their surface receptors and clones, and secrete many effectors to kill tumors in the microenvironment [75]. Tumor patients with lung cancer can induce immune dysfunction in CD8 + T cells and affect their immune function [77].

Increased numbers of MDSCs in patients with lung cancer may inhibit the generation of protective immune CD8^+^ T cells, and the highly expressed iNOS and L-arginase I (Arg-1) of MDSCs promote the downregulation of CD3ζ chain expression of CD8^+^ T cells and inhibit the immune function of CD8^+^ T cells [78]. MDSCs generate large quantities of ROS and peroxynitrite, and can also produce large amounts of NO, which can combine with ROS to form reactive nitrogen species (RNS). Moreover, it can interact with major histocompatibility complex class one (MHC-I) molecules, leading to a decrease in the response of CD8 + T cells to antigen-specific stimuli of tumors [79, 80]. The production of RNS can lead to post-translational modification of chemokine CCL2 and reduce the recruitment of CD8 + T cells in tumors [81], and inhibiting the generation of RNS can promote the function of CTLs [82]. Combining animal and in vitro experiments, studies have demonstrated that MDSCs in mouse tumors upregulated the expression of PD-L1 and secreted immunosuppressive cytokines due to hypoxia. MDSC-mediated inhibition of T-cell proliferation was enhanced in vivo, compared with that under normoxia [35]. Further, MDSCs upregulate PD-1 receptors on CD8^+^ T cells in vitro [5]. PD-1 receptor expression is a significant indicator of T-cell exhaustion. PD-1 interacts with its ligand, PD-L1, to reduce the proliferation and survival ability of CD8^+^ T cells and inhibit their secretion of cytokines, which in turn prevents CD8^+^ T cells from exerting their immune response, promotes resistance to ICIs, and promotes tumor progression. The oversecretion of IL-10 by MDSCs may inhibit the production of protective factors such as IL-2, IFN-γ, and IL-12 by CD8^+^ T cells, thereby inhibiting their proliferation, leading to impaired antitumor immunity, and inducing apoptosis of CD8^+^ T cells [50, 80]. There are contradictory claims regarding in the regulatory effect of IFN-γ on CD8^+^ T cells. CTLS may produce IFN-γ, which in turn may inhibit the immune function of CTLS [83], However, Wu et al. found that silymarin can reduce the level of IL-10 in the immune microenvironment of mice with lung cancer, as well as induce mRNA expression of nitric oxide synthase-2 (iNOS2), MMP9, and alginase -1 (Arg-1), further reducing the proportion of MDSCs and inhibiting their function. At the same time, IL-2 and IFN-γ levels were increased in mouse tumor sera, and the infiltration and function of CD8^+^ T cells were promoted [84], It has been demonstrated that interferon (IFN) promotes the increase of Treg cells and MDSCs in TME and is detrimental to the therapeutic effectiveness of PD-1 by inducing nitric oxide synthase 2 (NOS2) expression [33]. Schouppe demonstrated that MO-MDSC in the spleen could effectively reduce CTL-mediated antitumor immune function by increasing IFN-γ production, decreasing IL-2 responsiveness, affecting early CD8^+^ T-cell activation, reducing T-cell proliferation, and decreasing the expression of cytotoxic molecules [85].

Exosomes secreted by MDSCs deplete CD8 + T cells in mice. In vitro experiments have revealed that exosomes secreted by MDSCs can inhibit the proliferation of CD8^+^ T cells and induce apoptosis of CD8 + T cells by increasing ROS secretion and activating the Fas/Fas-L pathway [86]. This mechanism of action could involve iNOS expression and NO release, promoting CD8^+^ T-cell DNA damage and activating the p53 pathway [87]. In addition, MDSCs may influence the immune function of CD8^+^ T cells through transcription factors: Marigo's experimental study found that the immunomodulatory activity of MDSC was dependent on C/EBP-β transcription factors, and the immune tolerance of CD8^+^ T cells was reversed after C/EBP-β was cleared from the bone marrow of tumor mice [88].

### Interaction between MDSCs and NKT cells

**Fig. 1:**
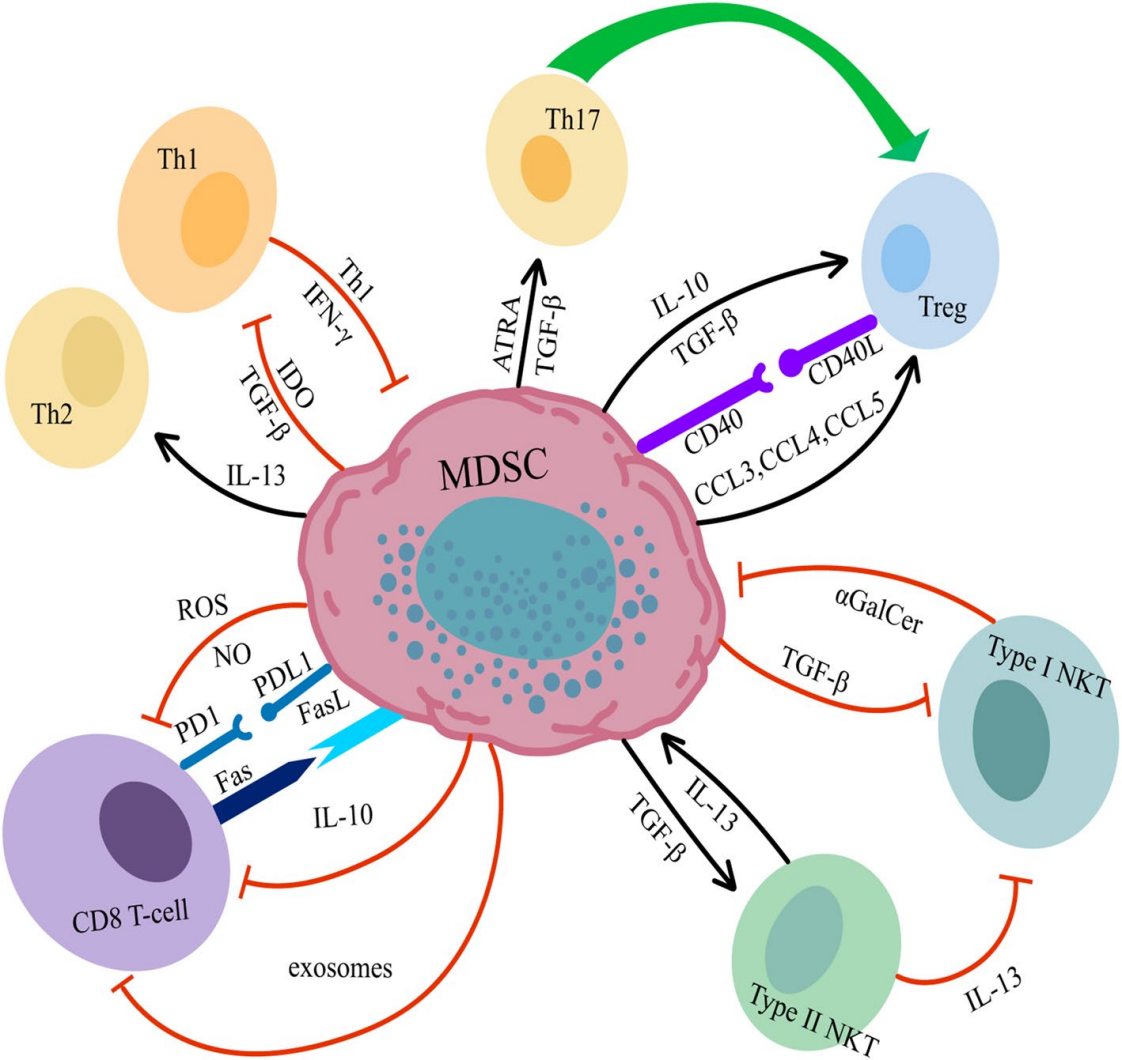
Interaction of MDSCs with T cells **a** MDSCs secrete IL-10 and TGF-β and express chemokines such as CCL3, CCL4 and CCL5 to promote the recruitment of Tregs; MDSCs express CD40 binding with CD40L on the surface of Tregs to promote the activation and expansion of Tregs. **b** The expression of IDO by MDSC inhibited the polarization of Th1 cells, and the production of TGF-β by MDSCs inhibited the function of Th1 cells. MDSCs promote Th2 cell differentiation through secretion of IL-13; MDSCs promote the transformation of Th17 cells into Treg cells by secreting TGF-β and RA. **c** MDSCs produce ROS and NO and secrete IL-10. Exosomes can inhibit CD8 + T cells’ function; MDSCs can upregulate PD-1 receptor on the surface of CD8 + T cells and promote CD8 exhaustion. **d** MDSCs inhibited the immune function of type I NKT cells by secreting TGF-β, and type II NKT cells inhibited the immune function of type I NKT cells by producing IL-13, and also induced MDSC to secrete TGF-β

NKT cells are a subpopulation of T cells that express both the TCR and NK cell receptor NKR-P1. NKT cells produce large numbers of cytokines and, like NK cells, NKT cells can exert cytotoxic effects. They play immunomodulatory roles in cancer, autoimmunity, allergies, infections, and other diseases. Similar to Th1/Th2 cells, NKT cells can be divided into type I NKT cells for protective immunity, type II NKT cells for suppressing immunity, and invariant NKT (iNKT) cells. The glycolipid ligand galactose ceramide (GalCer) activates iNKT cells in a cd1-dependent manner [89, 90], and can secrete cell death-inducing factors such as perforin and Fas/FasL. It can also activate and recruit a variety of antitumor effector cells to the TME, which have direct and indirect antitumor activities and can increase the antitumor immune function of patients with lung cancer [91], The increased expression of CD11b, CD40, CD11c, CD86, and MHC II on MDSCs results from the interaction of α-GalCer presented by MDSCs with iNKT cells, which can be converted to immunostimulatory APCs upon presentation with α-GalCer and tumor antigens [92]. The injection of iNKT cell agonists into mice with tumors led to a significant reduction in the frequency of MDSCs and the removal of the immunosuppressive factor IL-10 from MDSCs, leading to an increase in the frequency of tumor-specific T cells as well as inhibiting tumor progression [93]. The immunosuppressive function of MDSCs is abrogated by INKT cells in a CD1d-dependent fashion and can not only manipulate MDSCs but can also promote the acquisition of resistance to MDSCs by CD8 + T cells during their proliferation in vitro [94]. The main protective role played based on type I NKT cells in autoimmunity is produced by the production of IFN-γ, while MDSCs can selectively reduce the production of IFN-γ by NKT cells through membrane-bound TGF-β, thereby inhibiting the immune function of type I NKT cells. The capacity of the NKT cells to produce IFN-γ is also restored after the depletion of MDSCs [95]. Type II NKT cells are lipid-specific CD1d-restricted T cells that do not normally recognize-GalCer. Type II NKT cells upregulate IL-13 expression to inhibit CTL-mediated tumor immune surveillance and tumor-specific CD8^+^ T cells, which are involved in cancer progression [96]. Type II NKT cells can induce TGF-β secretion from MDSCs by producing IL-13 [97–99], and blocking IL-13 or TGF-β.Eliminating NKT or myeloid cells may interrupt this immunosuppressive circuit and uncover immune surveillance to prevent tumor recurrence [97] (Fig. 1).

### MDSCs promote T-cell exhaustion

T-cell exhaustion refers to the excessive activation of T cells in response to persistent antigenic stimulation, such as viral or tumor stimulation, the continuous co-expressing multiple inhibitory receptors on their surfaces, the inactivation of cellular effector effects, and the inability to differentiate into memory T cells and perform immune functions, such as tumor cell killing or virus clearance. The most significant features are the loss of effector cytotoxic functions (decreased secretion of the antitumor cytokines IL-2, IFN-γ, and TNF-α), leading to changes in the expression of key transcriptional factors, and the upregulation or coexpression of multiple inhibitory molecular receptors (such as PD-1, BTLA, CTLA4, TIM-3, LAG3, TIGIT) [100, 101], and most current studies focus on CD8^+^ effector T cells. Under normal immune conditions, immunosuppressive molecule receptors can be temporarily expressed on the surface of activated effector T cells, but are quickly downregulated as T cells are activated. However, these inhibitory molecules cannot be downregulated during T-cell exhaustion and continue being strongly expressed on the effector T-cell surface. T-cell exhaustion is one of the major factors affecting the efficacy of immunotherapy; it not only causes effector T cells to lose their antitumor immune function but also causes ICIs and other immunotherapy methods to lose their roles and targets [102]. Studies have shown that patients with lung cancer with a high expression of exhausted T cells have a worse prognosis, and improving the exhaustion status of T cells can improve the therapeutic effect in patients with lung cancer [103].

General control nonderepressible (GCN) is a serine/threonine kinase found in eukaryotic cells that may influence bone marrow function by regulating metabolism or protein production. It is a key driver of macrophage and MDSC polarization within the TME. Halaby found that the ability of GCN2 cKO tumors to express the T-cell exhaustion ligands CD206 and PD-L1 was significantly decreased. Simultaneously, the expression levels of PD-1 in CD4 + and CD8 + T cells decreased significantly. Flow cytometry showed that the expression of PD-1, LAG3, TIM3, and TIGIT on the surface of CD8 + T cells also decreased significantly. Thus, GCN, which promotes the polarization of MDSCs, can induce T-cell exhaustion [104].

MDSCs negatively correlate with CD8^+^ T-cell expression in lung cancer patients, and MDSCs can directly induce CD8^+^ T cells to express T-cell exhaustion inhibitory receptors such as PD-1, TIGIT, LAG3, CTLA4, and TIM3 [5, 105, 106]. MDSCs in the blood of NSCLC patients can inhibit the immune function of T cells, promote T-cell exhaustion, and affect the effectiveness of immunotherapy by expressing Galectin-9, a ligand of Tim-3 [23]. CD155, expressed as MDSCs binds to TIGIT on CD8^+^ T cells, deprives effector T cells of glucose utilization, reduces the expression on CD8^+^ T cells, and inhibits antitumor functions [107].

MDSCs can cause T-cell cycle arrest by secreting IDO. However, the application of IDO1-shRNA to inhibit the expression of IDO1 in lung cancer mice downregulates the expression of the inhibitory receptors PD-1 and BTLA on T cells, positively regulates the secretion of cytokines such as IL-2 and TNF-α, reverses T-cell exhaustion, delays the onset of tumors, and inhibits tumor growth [108]. Cytokines secreted by MDSCs such as IL-10 and TGF-β are also associated with T-cell failure. IL-10 is a STAT-3 inducing cytokine that normally attenuates T-cell activation. IL-10 can produce various downstream effects through the IL-10R-STAT3 signaling pathway. Blocking the IL-10R-STAT3 signaling pathway can change the chromatin of CD8 + T cells, thereby promoting the activation of CD8 + T cells and inhibiting their exhaustion [109]. IL-10 may directly affect T cells through the STAT-3 pathway, indirectly affect T cells through APC regulation of T cells, or both. TGF-β, like IL-10, can activate downstream SMAD transcription factors to further attenuate or inhibit immune cell activation. Enhancement of the TGF-β signaling pathway results in a significant decrease in the number of antigen-specific CD8^+^ T cells and in the production of antitumor cytokines, both of which are evidence of CD8^+^ T-cell exhaustion [110, 111] The attenuation of TGF-β signaling increases the amount of antigen-specific CD8^+^ Tex cells, thereby enhancing viral control [100].

MDSCs can also induce T cells exhaustion through an immunosuppressive microenvironment. MDSCs can induce primary CD4^+^ T cells to proliferate and differentiate into Tregs and release immunosuppressive factors like TGF-β, IL-10 and IDO [112]. Transcription factors like Blimp-1 and T-bet are regulated by MDSCs, and promote the expression of immunosuppressive molecule receptors in effector T cells [113], or inhibit effector T-cell immune function through direct cell–cell contact, and assist tumor cells in evading immunity.

**Fig. 2:**
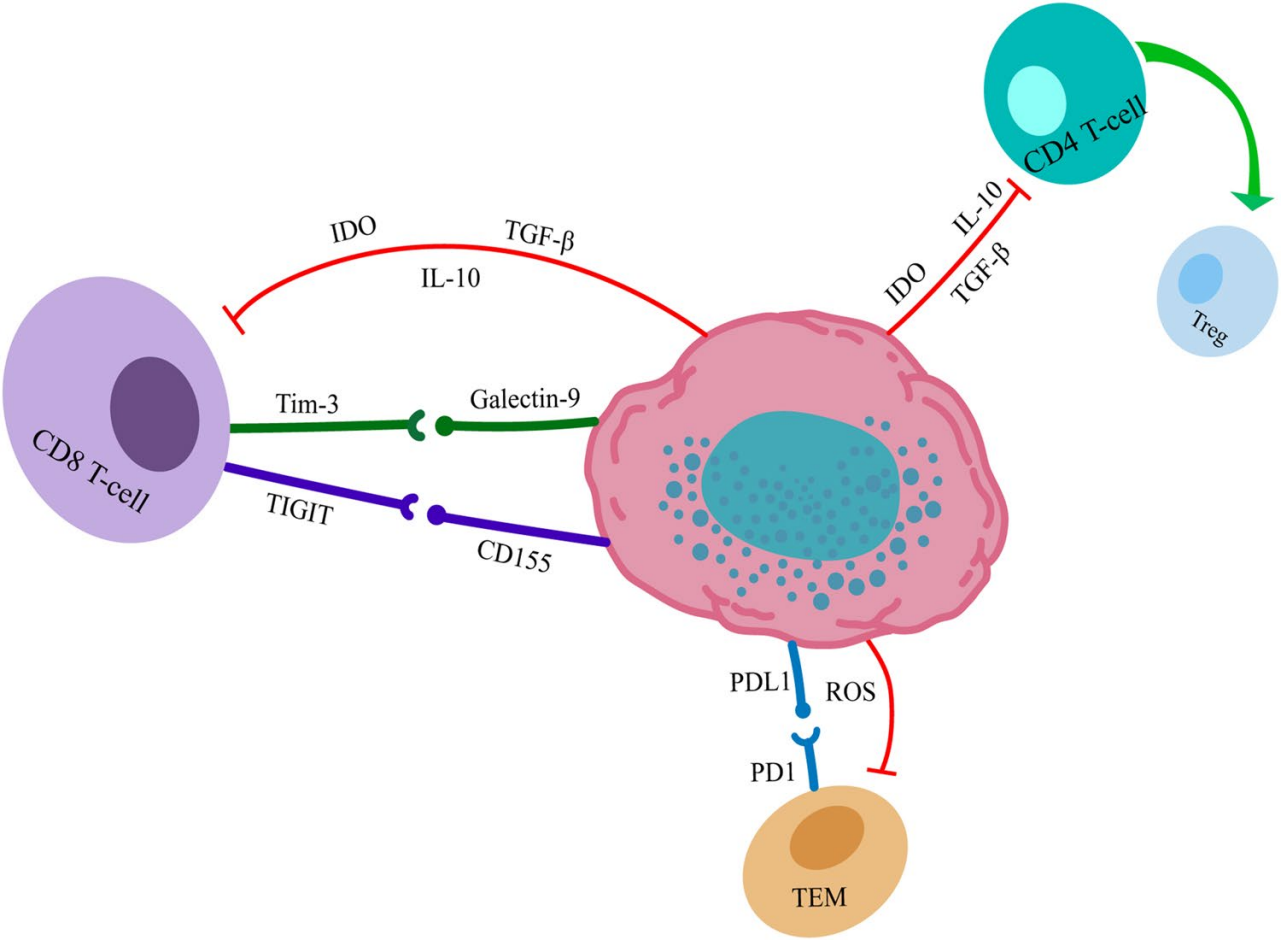
MDSCs and T-cell exhaustion. **a** MDSCs can induce CD8 + T cells to express T-cell exhaustion inhibitory receptors and secrete IDO, IL-10, and TGF-β to promote CD8 + T-cell exhaustion; **b** MDSCs can induce primary CD4 + T cells to differentiate and proliferate into Tregs; **c** MDSCs can induce PD-1 expression on Tem and promote CD4 + Tem depletion

Memory T-cell exhaustion is a new direction in tumor immunotherapy research. The degree of exhaustion of early memory T cells and T cells has been reported to correlate with the therapeutic efficacy of chimeric antigen receptor (CAR) T cells [114]. Reversing T-cell exhaustion can restore memory T -cells functions, improve the efficacy of CAR-T cell therapy, and enhance the inhibitory effects of CAR-T cells on solid tumors [115]. Antigen-specific CD4^+^ or CD8^+^ T cellsare activated upon exposure to antigens and can differentiate into effector and memory T cells. Effector T cells bind specifically to antigens and memory T cells, which persist as a heterogeneous population at multiple sites, and can coordinate protective immune responses to re-exposure. There are two subgroups of memory T cells: central memory T cells (Tcm) and effector memory T cells (Tem). Tcm is primarily located in secondary lymphoid organs and can rapidly proliferate and differentiate into effector T-cells in response to antigens. Tem cells are mainly located in the peripheral tissues and can rapidly produce effector cytokines once stimulated by antigens [116]. Both Tem and Tcm respond rapidly to antigens, generating a variety of effector molecules, and generating effector and memory cells. There are also lymphocytes called stem memory T cells (TSCM), which are associated with the naive-like surface marker TCF-1 high memory T cells and memory precursor-like T cells [117]. They have a higher proliferative capacity, reorganize the immunodeficient host more effectively, and mediate a superior antitumor response [118]. TSCM is considered suitable for adoptive T-cell immunotherapy [119]. The metabolism of CD4^+^ and CD8^+^ memory T cells in lung cancer patients contributes to long-term immunity [120]. However, the oxidative stress response caused by the increased ROS produced by MDSCs can produce related free radicals and immunomodulatory cytokines, which can inhibit host CD8^+^ and CD4^+^ T-cell responses, thus promoting the metastasis and the progression of lung cancer [121, 122]. Intratumor-infiltrating MDSCs are able to induce CD4^+^ TEM PD-1 expression, which promotes CD4^+^ TEM exhaustion [123]. When MDSCs and ROS are exhausted in the TME, the percentages of Tem, Tcm, and TSCM increase significantly and rapidly, the cytotoxicity and activity of memory CD8^+^ T cells increase, and the STAT-3 pathway can also be activated to maintain long-term memory and improve antitumor immune mechanisms [124] (Fig. 2).

### MDSCs inhibit T cells immune function by promoting EMT

EMT refers to the morphological transformation of epithelial cells to a fibroblast or mesenchymal phenotype in response to physiological or pathological stimuli. Loss of cell polarity, rearrangement of the cytoskeleton, increased migratory motility, and resistance to apoptosis are important processes that mediate tumor invasion and metastasis [99]. When MDSCs are co-cultured with tumor cells, cancer cells undergo morphological, behavioral, and phenotypic changes that are typical of EMT. MDSCs can mediate EMT in the tumor cells of various cancers, thereby promoting tumor progression. MDSCs in the lung cancer microenvironment can activate the AKT and ERK signaling pathways by highly expressing chemokines such as CCL11, which promote NSCLC cell invasion and induce EMT, thus further promoting NSCLC metastasis [125]. Li [126] found that MDSCs in contact with tumor cells can enhance the expression of Cyclooxygenase-2 (COX-2) in tumor cells, activate the β-catenin/TCF4 pathway, and promote the occurrence of EMT in tumor cells. However, reducing iNOS and TGF-β expression can reverse the promoting effects of MDSCs on EMT. In vitro experiments by Toh et al. [127] have found that the expression of epidermal growth factor (EGF), hepatocyte growth factor (HGF), and TGF-β1 in MDSCs were significantly upregulated after coculture with tumor cells, while the inhibitory effect of MDSCs on the EMT of tumor cells was significantly decreased after simultaneous application of inhibitors of the above three factors. The results of these studies demonstrate that EGF, HGF, and TGF-β1 play important roles in the induction of EMT in PMN-MDSCs. The IL-6/STAT3 signaling pathway can effectively trigger EMT and increase the number of tumor stem cells [128]. A study by Panni [129] has shown that M-MDSCs play a significant role in promoting tumor stemness and EMT by regulating the STAT3 pathway through the secretion of IL-6, and MDSCs also induce angiogenesis in a STAT3-dependent manner [106]. Studies have also confirmed that Ginsenoside Rg3 (Rg3) can effectively reduce the incidence of tumor cell stemness and the EMT by depleting MDSCs in the tumor and downregulating the STAT3 pathway [130].

**Fig. 3:**
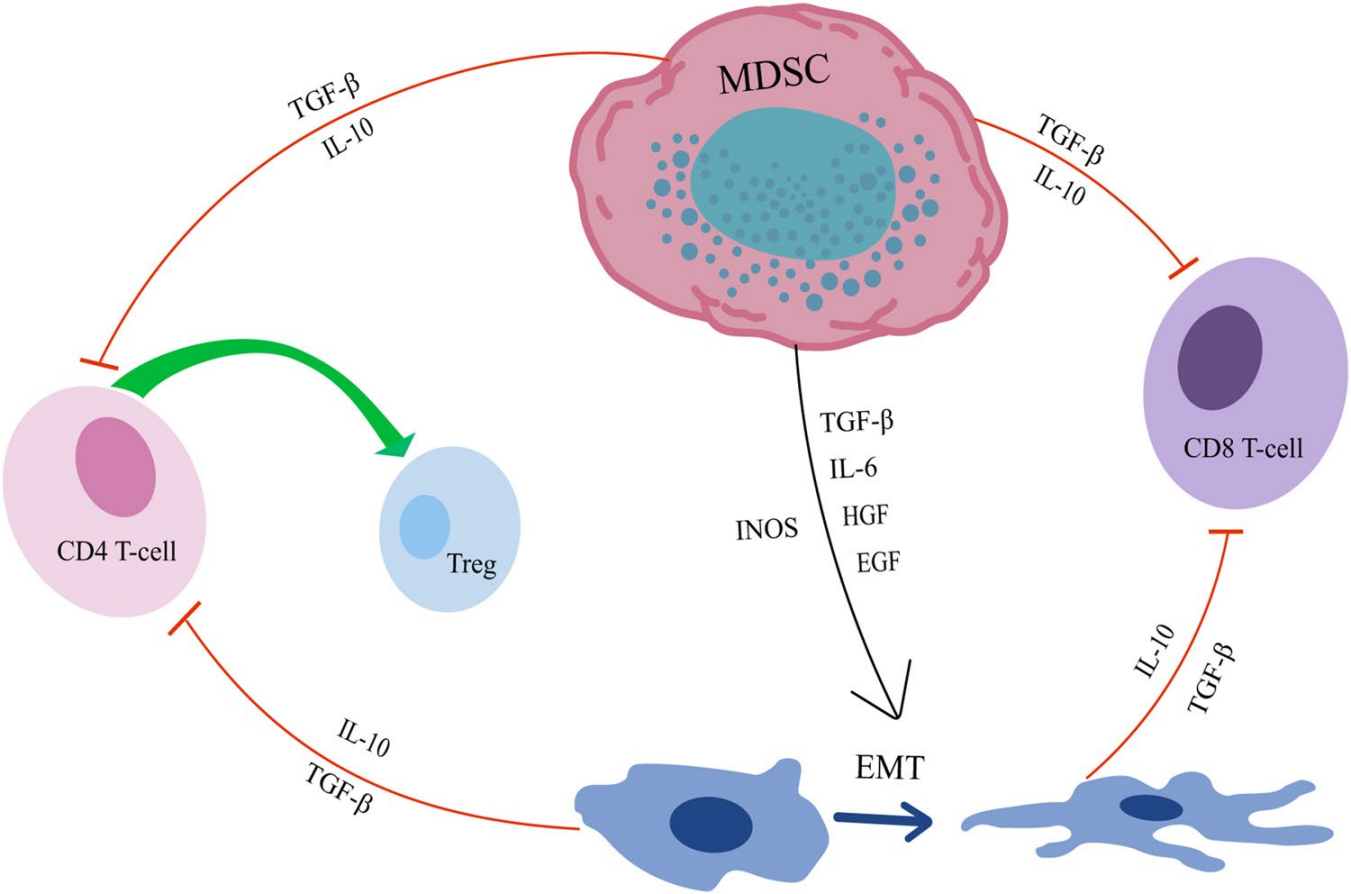
MDSCs and EMT **a** MDSCs inhibit the immune functions of CD4^+^ T and CD8^+^ T cells by secreting cytokines such as IL-10 and TGF-β; **b** Multiple factors secreted by MDSCs participate in the formation of EMT

MDSCs can inhibit T-cell immune functions in various ways, and the EMT process mediated by MDSCs is linked to the reduction of CD4^+^ and CD8^+^ T cells in the TME, recruitment of Tregs, and the exhaustion of T cells [131, 132]. The occurrence of EMT promotes the predominant metabolism of tumor cells in an aerobic glycolytic manner, which promotes the formation of an acidic tumor environment and the activation of HIF-1α, a transcription factor related to hypoxia [133], and the HIF-1α protein can participate in the recruitment of Tregs [49]. EMT-activated tumor cells express low MHC-I levels and elevated PD-L1 levels, exhaust CD8 + T cells, and recruit Treg cells [134]. EMT is closely linked to the inflammatory lung adenocarcinoma TME. In lung adenocarcinoma with the EMT phenotype, inflammatory factors were secreted and there was increased infiltration of multiple T-cell-exhausted immune checkpoint molecules, including the increased infiltration of PD-1, BTLA, CTLA-4, and TIM-3, as well as CD4 + Foxp3 + Tregs [135]. High levels of TGF-β and IL-10 expression can directly inhibit the immune function of CD4^+^ and CD8^+^ T cells and promote CD4^+^FOXP3^+^ Treg differentiation, inhibiting the function of Th1 cells [136–138], and blocking TGF-β, which increases the infiltration of CD8 + T cells into the TME and reduces the levels of MDSCs and Tregs [139]. In addition, EMT is closely related to the mechanisms of drug resistance that occur during lung cancer treatment [140, 141] (Fig. 3).

### Targeting MDSCs restores the immune function of T cells

Koh et al. used anti-Gr1 or anti-Ly6G to deplete MDSCs in mice with lung cancer; the activity and number of CD4^+^ and CD8^+^ T cells, memory T cells, and other immune cells were increased, and the secreted antitumor factors were also increased, which could promote the antitumor immune response [5, 6]. Studies have found that Resveratrol (RSV) can reduce the accumulation of MDSCs, promote their differentiation, induce the apoptosis of MDSCs, damage the inhibitory ability of CD8^+^ T cells, and improve antitumor immunity [142, 143]. Indomethacin (IND), a nonsteroidal anti-inflammatory drug, reduces tumor-induced MDSC accumulation, increases CD8^+^ T-cell infiltration, reduces Treg infiltration, alleviates chronic inflammation, and inhibits tumor progression and metastasis [144]. L-name, an iNOS inhibitor, can effectively reduce the recruitment and aggregation of MDSCs, and-galactosyl ceramide (GalCer) acts as a ligand for NKT cells. It enhances the host immune system by activating NKT cells. L-NAME combined with GalCer significantly increased the tumor antigen-specific response of mice with lung metastasis, increased the proportion of CD8^+^ T cells in the bronchoalveolar lavage fluid, and increased the antitumor effect of GalCer in a model of lung metastasis [145].

MDSCs immunosuppressive effect in the TME affects the long-term efficacy of lung cancer chemotherapy [146]. Gemcitabine combined with a superoxide dismutase (SOD) mimetic can reduce the activation of the IDO pathway, deplete MDSCs in mice with lung cancer, reduce Treg infiltration, inhibit CD8^+^ and CD4^+^ T-cell exhaustion, and improve the quantity and quality of memory T cells, to further promote the T-cell-mediated antitumor immune response and enhance the therapeutic efficacy of chemotherapy [124, 147].

ICIs have achieved some efficacy in NSCLC, and inhibitors targeting PD-L1 and/or PD-1 have been approved for use in NSCLC. Targeting PD-L1 and PD-1 increases tumor-specific T-cell immunity. PD-1/PD-L1 checkpoint inhibitors have demonstrated efficacy in advanced and refractory NSCLC in several clinical studies [148]. MDSCs can destroy the PD-1 therapy efficacy and depletion of MDSCs increases the amount of effector CD8^+^ T cells in multiple immune organs in mice, thereby enhancing the antitumor effect of anti-PD-1 treatment [149]. MEKi, CCL2 antagonists, entinostat, and other medications in conjunction with PD-1/PD-L1 mAbs can reduce the number of MDSCs, preventing differentiation of MDSCs, inhibiting immune function of MDSCs, and enhancing anti-PD-1 to induce an antitumor response. Moreover, it promotes antitumor immunity by enhancing CD4^+^ and CD8^+^ T-cell infiltration and inhibiting Treg cell expression (Table 3) [150–152].

**Table 3:** Target MDSCs to restore T cell immune function

Drug	Tumor model	Effector cells	Means of intervention	Results	Refs.
Individual antibody mediated depletion of MDSC (anti-Gr1 or anti-Ly6G)	3LL lung cancer mice model	CD8^+^T cells	Deplete MDSCs in mice	Increased APC and CD8^+^T cell activity	[5]
anti-Gr1 or anti-Ly6G	Lewis Lung Carcinoma (LLC) murine	CD8^+^T cells, Memory T cell	Depletion of MDSCs promotes the immune function of CD4^+^, CD8^+^T cells and memory T cells	Increased number of CD4^+^ and CD8^+^ T cells and increased secretion of anti-tumor factors by memory T cells	[6]
Resveratrol (RSV)	LLC murine	CD8^+^T cells	By inhibiting Arg-1 expression and ROS production reducing MDSC accumulation, inducing MDSC apoptosis, and promoting MDSC differentiation	Impaired the suppressive ability of MDSCs on CD8^+^T cells and promoted the expansion of CD8^+^IFN-γ + cells	[142]
Cimetidine	LLC mice	CD8^+^T cells	Reduce MDSCs accumulation and promote MDSCs apoptosis	Reversal of MDSCs-mediated T-cell suppression and promotion of IFN-γ secretion	[143]
indomethacin (IND)	LP07 murine	Treg、CD8^+^T cells	Reduced arginine activity, inhibited NO and ROS production in MDSCs, and thus suppressed immune function in MDSCs	Increased infiltration of CD8^+^T cells and decreased infiltration of Treg	[144]
Alpha-garactosylceramide (GalCer) + L-NAME	Mice with lung metastases	CD8^+^T cells、NKTcells	Inhibit iNOS activity and reduce the recruitment and aggregation of MDSCs	Activate NKT, increase the number of CD8 + T cells	[145]
Gemcitabine (Gem) and a Superoxide dismutase mimetic (SOD mim)	LLC murine	memory CD8^+^T cells,Treg	Depletion of MDSCs and their ROS production in mice activates memory CD8^+^ T cells of STAT-3 signaling pathway	Increase the quantity and quality of memory CD8^+^ T cells and reduce Treg infiltration	[124]
Gemcitabine (GEM) and a SOD mimetic (SOD)	LLC mice	CD8^+^, CD4^+^T cells, TCM, TSCM	Reduce MDSC IDO pathway activation	Suppression of CD8 + and CD4 + T cell exhaustion, increased percentage of CD8 + T lymphocytes in TCM and TSCM	[147]
all-trans retinoic acid (ATRA) and wild-type p53 vaccination	Patients with SCLC	CD8^+^T cells	Depletion of MDSCs cells and promotion of anti-tumor immune response	Increased proportion of CD8^+^ T cells	[158]
MEKi and anti-PD-1/PD-L1 mAbs	PKL5–2 murine lung tumor	CD8^+^ T cells, CD4^+^ T cells	Reduced the number of MDSCs, prevented the differentiation of MDSCs, and enhanced the anti-PD-1-induced anti-tumor response	Increase the number of CD8 + T cells, CD4 + T cells cells in the tumor microenvironment	[150]
CCL2 antagonist and anti-PD1 antibody	LLC murine	CD8^+^ T cells, CD4^+^ T cells	Inhibits Arg-1 and iNOS protein expression, reduces the recruitment of MDSCs, and enhances anti-PD-1-induced antitumor response	Increased infiltration of CD4^+^ and CD8^+^ T cells	[151]
Entinostat + anti-PD1 antibody	LLC mice	Treg、CD8^+^ T cells	Suppression of immune function in MDSCs and enhancement of anti-PD-1-induced antitumor response	Attenuate the inhibitory effect on CD8^+^ T cells and suppress Treg cell function	[152]

## Clinical application of targeting MDSCs to reshape lung cancer immune microenvironment

Products that target MDSCs have also been shown to have inhibitory effects on the number and function of MDSCs. One of the most prominent features of MDSCs is ARG1. In recent years, vaccination for antibodies against ARG1, coupled with inhibitors of the immune checkpoint, have been found to increase T-cell infiltration, restore the function of CD8 + T cells, and promote antitumor immune responses [153, 154]. Clinical evidence suggests that MDSCs, like anti-PD-1/PD-L1 and anti-CTLA-4, can weaken the response to immunotherapy. Tracking the dynamics of MDSCs during immunotherapy and controlling their expansion, recruitment, and function in tumors are crucial for treating immunotherapy-resistant patients [155]. ROS are also one of the products that influence the function of the MDSCs; the application of mitochondria-targeted and ultrasound-responsive PIO-NH nanoparticles can effectively improve hypoxia, inhibit ROS production, decrease the level of MDSCs in tumors, increase CD8^+^ T cell activity, activate the antitumor immune response, and inhibit metastasis [156]. There are relatively few clinical trials directly targeting MDSCs; however, clinical trials have investigated whether different drugs can decrease the number of MDSCs in peripheral blood from lung cancer patients (Table 4). PD-1 blockers can effectively eliminate MDSCs from the peripheral blood of NSCLC patients. Gemcitabine combined with the PD-1 blocker nivolumab can also effectively reduce MDSC levels in the peripheral blood from patients with NSCLC. All-trans retinoic acid (ATRA), which is used as a therapeutic compound targeting MDSCs, can deplete MDSCs in the TME [157]. Clinical studies have confirmed that patients with extensive-stage SCLC treated with ATRA and vaccinated with wild-type p53-transduced DC vaccines can effectively deplete MDSCs, increase the proportion of immune CD8^+^ T cells, promote antitumor immune responses, and enhance the effect of chemotherapy [158]. Currently, the number of clinical studies on MDSCs is relatively small and the number of patients included in the existing studies are relatively few. Therefore, multicenter studies with large sample sizes are required for more definitive results.

**Table4:** Clinical study of targeting MDSCs

Drug	Paitents	Clinical development status	Experimental design	Conclusion
Gem	Patients with stage IIIB NSCLC	Phase 2 experiment (NCT03302247)	To evaluate whether Gem can improve the efficacy of nivorumab by reducing tumor immune suppression by targeting MDSCs	Gem can reduce MDSCs and improve T cell activity in peripheral blood of NSCLC patients
Nivolumab	NSCLC	Completed (NCT03486119)	To evaluate the dynamic changes of immune cells in peripheral blood of NSCLC patients during treatment with nivorumab, a PD-1 blocker	After one cycle of treatment, the proportion of CD11b^+^CD33^+^MDSCs in peripheral blood of NSCLC patients decreased significantly
Receptor Antagonist, AAT-007 (RQ-07; CJ-023,423) + Gem	NSCLC	Phase 2 Experiment (NCT02538432)	To evaluate the dynamic changes of MDSCs in peripheral blood of patients after AAT-007 intervention	NA
All-Trans Retinoic Acid (ATRA) and atezolizumab	Advanced NSCLC	Phase 1 Experiment (NCT04919369)	To investigate the effect of ATRA on the level of MDSCs in peripheral blood of patients	NA
PBF-1129 and nivolumab	Advanced NSCLC	Phase 1 Experiment (NCT05234307)	To investigate the effect of PBF-1129 on TME and MDSCs levels in peripheral blood of patients	NA
Sargramostim plus pembrolizumab with or without pemetrexed	Advanced NSCLC	Phase 2 experiment (NCT04856176)	The changes of MDSCs and CD4^+^, CD8^+^T cells at different time points during the treatment were evaluated	NA

## Conclusions

In general, MDSCs in the peripheral blood and tumor tissues of lung cancer patients are often higher than those of healthy people. MDSCs inhibit the T-cell immune functions by upregulating the immunosuppressive pathway and secreting immunosuppressive molecules and chemokines, which promote the immunosuppression and treatment resistance of lung cancer patients, which then leads to tumor progression and metastasis. Therefore, MDSCs have been identified as a major impediment in treating patients with lung cancer, and the depletion of MDSCs in the lung cancer microenvironment has certain clinical implications for the treatment of lung cancer. Drugs targeting MDSCs have been developed, but there are still many open questions about their mode and mechanism. The mechanism of relevant drugs should be clarified in future experiments to ensure positive results in all lung cancer patient populations.

## Data Availability

Not applicable.

## Abbreviations

APCs: Antigen-presenting cells
ARG1: Arginase 1
ATRA: All-trans retinoic acid
CD40L: CD40 ligand
CTL: Cytotoxic T lymphocyte
COX-2: Cyclooxygenase-2
EGF: Epidermal growth factor
EMT: Epithelial mesenchymal transition
GalCer: α-Garactosyl ceramide
HGF: Hepatocyte growth factor
HIF-1α: Hypoxia inducible factor-1α
IDO: Indoleamine 2,3-dioxygenase
IMC: Immature myeloid cells
IND: Indomethacin
iNOS: Inducible nitric oxide synthase
MDSCs: Myeloid-derived suppressor cells
MHC-I: Major histocompatibility complex class one
M-MDSCs: Monocytic MDSCs
NKT cell: Natural killer T cells
NO: Nitric oxide
NSCLC: Non-small cell lung cancer
PD-1: Programmed death factor-1
PD-L1: Programmed death ligand-1
PMN-MDSCs: Polymorphonuclear MDSCs
Rg3: Ginsenoside Rg3
RNS: Reactive nitrogen species
ROS: Reactive oxygen species
RSV: Resveratrol
SCLC: Small cell lung cancer
SOD: Superoxide dismutase
STAT3: Signal transducers and activators of transcription3
TCM: Central memory T cells
TCR: T cell receptor
TEM: Effector memory T cells
Th cell: T helper cells
Treg: Regulatory T cells
TSCM: Stem memory T cells
αGalCer: α-Galactosylceramide
